# Proceedings at General Meeting

**Published:** 1914-07-11

**Authors:** 


### NATIONAL MEDICAL UNION (Official).

### Proceedings at General Meeting.

A General Meeting of the National Medical Union was held at the Café Monico, Piccadilly Circus, London, W., on Saturday, June 27, Dr. V. T. Greenyer, F.R.C.S., Brighton, in the Chair.

The Report of the Provisional Federating Council was discussed.

On the motion of Dr. Oppenheimer the Treasurer's report was approved.

In connection with the discussion Dr Carswell drew attention to the recommendation that as soon as the funds permitted the services of a paid business manager should be secured. He said that owing to the necessity of paying close attention to his own private practice, it would be impossible for him to continue to give the same amount of time to the affairs of the Union as he had done in the past; and, as it was imperative not only that a similar amount of work should be performed, but even more, he had decided with regret to resign from the position of honorary secretary in favour of a general secretary, who should receive an honorarium for his services. As had been previously intimated, both the present secretaries intended that any services which they might perform should be purely honorary; and the best arrangement that he could suggest would be that Mr. Dorrell should remain honorary secretary, and that the general secretarial work should be performed by a whole or part time paid business manager.

The adoption was agreed to of section (2) of the Report of the Federating Council, printed in The Hospital, June 6, 1914, p. 278.

Dr. Porter proposed that the National Medical Union should apply for registration under the Companies Act, 1867, and that the liability of members should be, by guarantee, £1. This was carried.

Dr. Playfair moved: "That the permanent officers should be two honorary presidents, not less than three honorary vice-presidents, secretaries, treasurers, and Council, with committees, executive, organising, finance, Parliamentary, and Press. This was carried.

Dr. Blake proposed the adoption of sub-section (3). This was carried.

The Chairman moved sub-section (4) of the same Report. To this Dr. Playfair moved an amendment that the condition should be made provisional, being made to read: "That until the next General Meeting every Association," etc. This amendment was carried.

Dr. Porter moved that where a branch is entitled to more than one member of the Council of the National Medical Union, any one member of Council attending any meeting of the National Medical Union should be empowered to represent the whole of the members of the branch in the absence of any other members of the Council. This should be incorporated in the rules of the Association.

After a great deal of discussion an amendment that the question be adjourned until the next annual general meeting was carried by fourteen to eleven.

Sub-section (5) of the same Report was carried.

Dr. B. G. Morison moved: "That the National Medical Union being now regularly constituted and having a definite course of action, it is advisable to issue a public statement of its attitude with regard to the alterations introduced into medical practice by the National Insurance Acts, and that the Council take the necessary steps for this purpose."

Dr. A. F. Shoyer (Islington and St. Pancras) seconded this resolution, at the same time moving the addition of the following words: "and that a form of declaration be drawn up and printed for signature by insured persons who do or intend to pay the private doctor of their choice rather than consult a panel doctor."

Dr. Morison accepted the addition. Thus amended the resolution was put to the meeting and carried unanimously.

On Section (4) of the Report Dr. Playfair moved that Paragraph 1 be adopted, but ending at the word "freedom," so as to read: "To organise the medical profession to the highest possible degree of efficiency in defence of its traditional freedom."

This was lost, and the paragraph was adopted as originally proposed.

Paragraph 2 was amended to read: "To render every service possible to non-panel practitioners." As thus amended it was agreed to.

Paragraphs 3, 4, 5, and 6 were agreed to.

In Paragraph 7 the word "necessitous" was substituted for the word "poor," and in this form the resolution was agreed to.

In Paragraph 8 the word "voluntary" was deleted, and thus amended the paragraph was agreed to.

Dr. Greene moved: "That refusal of service in any capacity under the existing National Insurance Acts, 1911 and 1913, be incorporated as a primary principle in the policy of the National Medical Union." He said this non-service resolution expressed three or four things. One was non-service on Insurance Committees. Doctors had now had time to sit quietly by and chafe under the working of those committees. They had seen them in some cases advocate the legalisation of unqualified herbalist practitioners. From the very start those committees had been against the free choice of doctors. Out of fifteen and a-half millions insured persons less than two thousand had been allowed to make their own arrangements in this matter. The panel men should be left to work their panels.

Another thing the resolution would do would be to prevent members of the Union becoming referees. In Ireland a dispute as to the terms on which the certificate of fitness for employment should be signed had led to the Government appointing their own referees to follow round after the usual attendant. One of these had certified a man fit for work who died of cancer two days after. Another case was that where the certificate was given though the man was suffering from aortic aneurism.

A long discussion followed. An amendment of Dr. Brassey Brierley, seconded by Dr. Porter, was accepted by the mover and seconder of the resolution. Thus amended it read: "That refusal of service in any capacity under the existing National Insurance Acts, 1911 and 1913, be a condition of membership of the National Medical Union."

Dr. Macnamara thought the rein was being drawn too tight. He had been in public life, and might be again. If a public body on which he was a representative invited him to become a member of a committee dealing with the Insurance Acts where there was no fiduciary benefit to himself at all, he would have to consider whether his primary duty was to his constituents or to the National Medical Union. He believed doctors on such a committee might do excellent work alike for the profession and the public so long as they were not on the panel and not accepting service at the expense of the State.

Dr. Morison supported this view. He thought the words "in any capacity" should be left out of the resolution. Many others having spoken, Dr. Oppenheimer moved that the matter be left to local autonomy. Dr. Handson seconded this. He said the question was of the very greatest importance. It had very nearly ruined the chance of the National Medical Union being formed in November last. It made a fundamental difference that in some places—*e.g.*, Edinburgh—they had started on the principle of having absolutely nothing to do with the Act, whereas in London a different policy had been followed.

This amendment having been debated at length, nine voted for the amendment and twenty-five against.

Another amendment was moved by Dr. Carswell: "That refusal of service in any capacity under the existing National Insurance Acts be a unanimous recommendation of the general meeting to all non-panel practitioners."

At the request of Dr. Oppenheimer the word "unanimous" was replaced by the word "strong."

Thus amended it was discussed, and at length lost, ten votes being cast for it and twenty-two against.

An amendment of Dr. Macnamara to delete the words "in any capacity" from the resolution was seconded by Dr. Morison, voted on, and lost, the voting being nine for and twenty-three against.

Mr. Dorrell asked Dr. Greene if he would consent to the following words being added to the resolution: "Nothing in this resolution shall prevent any member of the National Medical Union from signing certificates to enable insured persons to obtain sanatorium and sickness benefits under the National Insurance Acts in his private professional capacity."

Dr. Greene did not at first consent to the addition, and Mr. Dorrell moved it as an amendment. Dr. Macnamara seconded the amendment. Afterwards Dr. Greene consented to the addition. As thus amended the resolution read:—

"That refusal of service in any capacity under the National Insurance Acts, 1911 and 1913, be a condition of membership of the National Medical Union. Nothing in this resolution shall prevent any member of the National Medical Union from signing certificates to enable insured persons to obtain sanatorium and sickness benefits under the National Insurance Acts in his private professional capacity." As thus amended the resolution was carried by twenty-six votes to four.

The paragraph of the report relating to Form 43 I.C. was then discussed. On the motion of Dr. Porter, this was agreed to on the understanding that the recommendation should not be retrospective.

Paragraph 4 in the Resolutions on Policy was agreed to without discussion.

The paragraph of the report headed "Seamen's National Insurance Society" was then agreed to.

Dr. Johnston moved, and Dr. Brassey Brierley seconded: "That a guarantee fund be established to strengthen the financial position of the National Medical Union."

This was put to the meeting by the Chairman and agreed to.

Dr. Porter proposed the appointment of a general secretary at an honorarium of fifty guineas per annum. Dr. Johnston proposed that the appointment be left to the Council. Dr. Carswell seconded this, adding that it should be the first business of the Council. Dr. Johnston accepted this addition to his motion, and in this form the resolution was agreed to.

Dr. Carswell proposed that the two provisional presidents should be the two permanent presidents. Dr. Playfair raised the question of whether Professor Russell would continue to sit; and it was decided, on the motion of Dr. Carswell, seconded by Dr. Oppenheimer, "That the meeting request Dr. Playfair to express its unanimous hope that Dr. Russell would continue president."

Dr. Johnston moved, and Dr. Playfair seconded: "That the Council be authorised, if necessary, to appoint a president until the next general meeting." This was agreed to.

Dr. Porter moved, and Dr. Barton seconded, "That the number of vice-presidents be increased to six." This was agreed to.

The three retiring vice-presidents were re-elected. Dr. James Oliver and Dr. Brassey Brierley were appointed vice-presidents.

On a motion to appoint Dr. F. J. Smith also, that gentleman said that by a resolution passed that afternoon he was debarred from membership of the National Medical Union because the hospital men in London had elected him to serve on the Local Medical Committee for London. He had now before that committee a resolution dealing with the unallotted funds. He expected his resolution to be very heavily defeated, and that would be his opportunity to resign.

Dr. Greene moved, and Dr. Worth seconded: "That a place be kept warm for Dr. F. J. Smith as vice-president of the National Medical Union." This was put to the meeting and carried.

Mr. Dorrell was appointed honorary secretary.

On the motion of Dr. Brassey Brierley, seconded by Mr. Dorrell, a vote of thanks was heartily accorded to Dr. Carswell for his services.

Dr. Oppenheimer and Dr. Brassey Brierley having terminated their office as honorary treasurers, it was agreed that the election of a treasurer be left to the Council.

It was also agreed to appoint an audit committee to audit the accounts before they were passed over to the Council.

Dr. Greenyer, Mr. Dorrell, and Dr. Carswell were appointed to this committee.

Dr. Carswell moved a vote of thanks to the Chairman. It was heartily accorded, and the Chairman having briefly acknowledged the compliment, the proceedings terminated.

